# Baltimore Academy of Medicine

**Published:** 1886-01

**Authors:** 


### BALTIMORE ACADEMY OF MEDICINE.

Reported for THE ATLANTA MEDICAL AND SURGICAL JOURNAL.

STATED MEETING HELD DECEMBER 1ST, 1885.

The President, Dr. J. J. Chisolm, in the chair.

After the regular order of business the following cases were related :

### EVISCERATION OF EYE-BALL BY THE NEW METHOD.

Dr. J. J. Chisolm related a case of evisceration of the eye-ball after the plan recently recommended. The operation consists in completely excising the cornea by means of a circular incision around its margin. The contents of the ball are then to be entirely removed leaving the sclerotic intact. The advantage claimed for the operation is that the socket tissues are not disturbed, neither is the muscular apparatus of the eye interfered with ; besides, the stump left, after cicatrization leaves an admirable seat upon which to locate the artificial eye. The operation itself is a very simple one and can be performed much more expeditiously than can complete enucleation, but convalescence is so very tedious, and at times gives rise to such painful and alarming symptoms, as occurred in his case, that in future he will confine himself to the old plan of complete enucleation.

Dr. Chisolm said that it was his usual custom to allow a patient to go about his affairs very soon after the operation, at the outside, twenty-four hours, but in the evisceration, operation, even up to the fourth day and later, there was such œdema and pain that he could not think of allowing the patient to be from under his observation. He had never had such an experience with the old method.

Dr. S. C. Chew wished to know if sufficient anæsthesia could be produced, by the use of cocaine, to enable one to perform this operation without painful sensations on the part of the patient.

Dr. Chisolm thought not.

PARALYSIS, THE RESULT OF A FALL.

Dr. A. B. Arnold related a case which occurred at Bay View Asylum a year ago. It occurred in a woman five months pregnant. She fell from the second story window, striking upon the head. When picked up she was senseless, and remained so for three days. Among the consequences of the fall, abortion was brought on. At present she has paralysis of both lower extremities, complete anæsthesia on right side, is deaf, and also blind on this side.

She evidently has some spinal trouble, as she complains of that sensation so common to these troubles, "as if a belt were being drawn about her waist." Also has some enfeeblement of the sphincters of bladder and rectum. In addition, there is a peculiar nervous jerking about the head, which has continued for one year.

Dr. Arnold thinks the lesion is located about the inferior third of the internal capsule, extending up and involving the optic and auditory nerves at their point of crossing, and also does he take the nerves of sensation, which come off at this point, to be affected. He thinks the symptoms justify this conclusion. The pupils responded normally to light. She had never had any trouble previous to the fall.

Dr. Chisolm asked if any ophthalmoscopic examination had been made. He said he was prompted to ask this question by a case which recently came under his observation. It occurred in a man who had received a severe beating, and among the results, loss of vision began after about six weeks.

He examined him, and found anæsthesia of the fifth nerve, the hearing involved, and several small retinal hemorrhages around the point of entrance of optic nerve.

Dr. S. C. Chew related an interesting

### CASE OF EMPYEMA.

He had been called in consultation to see a case which occurred in a patient who for several days past had suffered from severe dyspnœa. The night before he saw the patient, he had had copious expectoration of matter, the nature of which he could not describe, as it was not saved. This was followed by immediate relief from the difficulty of respiration.

Upon physical examination of the chest, the left pleura was found to be about two-thirds filled with fluid. Shortly after his visit, the patient suddenly died with profuse vomiting of purulent matter, as well as the passage of quantities of it from the bowels. From such symptoms, he thinks it highly probable that perforation took place through the diaphragm into the stomach, and that the fluid was discharged in both directions through the œsophagus and through the intestines. He had searched the literature on the subject, and had found no case terminating in this manner recorded. Physical examination of chest, made before the first expectoration of matter, showed the pleural cavity to be entirely filled with fluid. More recently, he had seen a patient at the Baltimore Infirmary whose physiognomy was indicative of pulmonary trouble. Percussion over right side of chest gave decided resonance both anteriorly and posteriorly. On auscultation on same side there was pronounced *Hippocratic Succussion*. Shaking the patient while ear was still on the chest gave a sound very similar to that heard when one shakes a jug partly filled with water. No discomfort at all was noticed, neither pain nor dyspnœa. He thought the absence of dyspnœa could be explained on the ground that the air probably distended the pleura (and this compressed the lung) so gradually that this lung, as well as the healthy one, had sufficient time to accustom themselves to their abnormal relations. Whether perforation was due to ulceration outward through the pleura, from the wall of a cavity in the lung, or whether it was due to a loss of substance occasioned by a point of inflammation upon the visceral pleura, and this perforation taking place in the reverse direction, he could not say, although symptoms sufficiently marked were present to point to either mode.

In his opinion surgical interference should be resorted to as soon as the condition of empyema is made out.

Dr. Arnold had seen a case of empyema in a child. It occurred on the right side, ulcerated through the diaphragm, and discharged its contents into the abdominal cavity. The child died from shock. He considered it somewhat remarkable how rarely phthisical patients showed any signs of discomfort from interference with respiration, even though their lungs might be riddled with cavities.

Dr. Chew looked upon this as due to the slowness of the pathological process, thus enabling that portion of the lung, which is healthy, to increase its physiological activity. He also thought, in view of the fact that cavities so frequently form near the pleura, that it was singular that we so rarely found perforations from them.

Dr. Chisolm said serous sacs resisted perforation.

### THE COCAINE HABIT.

Dr. J. C. Thomas said since he had been told that in some of our principal cities the cocaine habit was largely being acquired, he would like to ask if any member had ever seen a case of it, and if the report is true, did not the gentlemen think that the medical profession should take some steps to prevent the reckless prescription of this seductive drug.

Dr. Chisolm related two cases in which decided loquacity was produced by the introduction of cocaine into a cavity in a decayed tooth. In speaking of cocaine tablets, he referred to a condition amounting almost to a slough, that was produced on the buccal surface of his own cheek from the application of one of these tablets to the gum and allowing it to remain until dissolved. From this he hardly considered them the thing to use in nasal catarrh.

